# The effect of hand massage on pain, comfort, and sleep quality in palliative care oncology patients

**DOI:** 10.1007/s00520-026-10558-5

**Published:** 2026-03-12

**Authors:** Murat Koc, Nurdan Yalcin Atar

**Affiliations:** 1 Pendik State Hospital, Istanbul, Türkiye; 2 Department of Fundamentals of Nursing, Hamidiye Faculty of Nursing, University of Health Sciences, Istanbul, Türkiye

**Keywords:** Palliative care, Oncology, Hand massage, Pain, Sleep quality, Comfort

## Abstract

**Objective:**

Oncology patients often experience pain, sleep problems, and discomfort during palliative care. Hand massage is a nonpharmacological method that can relax the patient and help alleviate these problems. This study aimed to examine the effect of hand massage on pain, comfort, and sleep quality in palliative oncology patients.

**Materials and methods:**

A randomized, controlled experimental study was conducted with a sample of 76 oncology patients treated in the palliative care clinic of a public hospital. The patients were randomly allocated to the experimental and control groups. The experimental group (*n* = 38) received a total of 16 sessions of hand massage, performed twice a day, 2 days per week for 4 weeks. The control group (*n* = 38) received no intervention. The patients’ pain, sleep quality, and comfort were assessed before and at 1, 2, and 4 weeks after the start of the intervention using the Visual Analog Scale for pain, the Pittsburg Sleep Quality Index, the General Comfort Questionnaire, and a smart wristband.

**Results:**

Demographic characteristics and pre-intervention pain, sleep quality, and comfort scores showed no statistical differences between the groups (*p* > 0.05). The experimental group reported significantly lower pain intensity and greater comfort than the control group starting from week 2 (*p* < 0.05). In addition, subjective sleep quality assessed using the PSQI and objective sleep parameters (sleep duration and sleep score) measured by a smart wristband, including objective sleep duration and objective sleep score, were significantly better in the experimental group than in the control group from the first week (*p* < 0.05).

**Conclusion:**

Hand massage is an effective method for reducing pain and increasing sleep quality and comfort in palliative oncology patients.

**Clinical trial registration no:** NCT06360614.

## Introduction

Globally, cancer is the second most common disease and leading cause of death [1]. The International Agency for Research on Cancer (IARC) predicts approximately 12 million new cases of cancer will be diagnosed worldwide by 2045 [2]. Depending on the disease and treatment process, cancer patients often experience symptoms such as pain, sleep disorders, and discomfort, all of which can seriously impair their quality of life. Palliative care aims to improve symptom management and quality of life, and is thus an indispensable part of cancer patient care. American Society of Clinical Oncology (ASCO) and National Comprehensive Cancer Network (NCCN) guidelines recommend that oncology patients receive palliative care concurrently with active treatment, and the World Health Organization (WHO) also emphasizes that palliative care is not only for the late stages of life but should be implemented from the time of diagnosis [2–5]. Nevertheless, in routine clinical practice, palliative care is most commonly delivered to patients with advanced-stage cancer, particularly in end-of-life settings [6].

The most distinct feature of cancer patients receiving palliative care is their multidimensional symptom burden. They experience myriad symptoms simultaneously, both during the treatment process and in the terminal stage [7]. Depending on the disease process, symptoms such as pain, loss of appetite, fatigue, nausea, and shortness of breath severely restrict daily life and can be accompanied by psychological problems such as sleep disorders, anxiety, and depression. Cancer patients also require support from family members and professional caregivers because of frequent hospital visits and intensive treatment [8].

Within the multidimensional symptom burden, pain and sleep disorders are the most common problems reported by most palliative care oncology patients. More than 80% of patients have pain [9] and approximately 60% experience a deterioration in sleep quality [10]. Pain is not merely a physical symptom, but a multidimensional phenomenon that limits activities of daily living, reduces independence, and increases care needs. Pain can trigger psychological problems such as unease, apprehension, and depression, leading to social isolation and negatively affecting sleep quality [11]. This deterioration in sleep quality can cause additional problems such as fatigue, distraction, anxiety, stress, a weakened immune system, and increased pain severity [12, 13]. There is a bidirectional relationship between pain and sleep quality; insufficient sleep reduces the pain threshold, and pain disrupts sleep quality, leading to a vicious circle [14]. This situation negatively affects not only physical but also emotional and mental comfort. Therefore, in the palliative care process, pain management, improving sleep quality, and supporting overall comfort are among the main priorities that must be addressed through a holistic approach [15].

In palliative care, both pharmacological and nonpharmacological methods are used in cancer symptom management [16]. Among the nonpharmacological methods, hand massage has an important place in palliative care due to its easy applicability and favorable effects [17]. Hand massage mechanically and neurologically stimulates the skin, muscles, internal organs, and circulatory and lymphatic systems [18]. Its effects can be classified at the physical, psychological, and physiological levels. The physical effects include promoting cellular regeneration and facilitating the removal of accumulated toxins, while the psychological effects include providing relaxation, supporting stress and anxiety control, and creating general well-being. The physiological effects of hand massage include reducing pain by increasing endorphin release, regulating circulation and respiratory function, strengthening the immune system, and increasing overall comfort and sleep quality [19–21].

Although there are studies in the literature examining the effects of hand massage on various variables in different sample groups [22–24], few have evaluated its effectiveness in cancer patients [25]. There are no studies specifically evaluating the effects of hand massage on pain, comfort, and sleep in oncology patients receiving palliative care. This study aimed to eliminate this gap in the literature and examine the effects of hand massage on pain, comfort, and sleep in this patient group.

### Research hypotheses

- **H**_**1**_**:** Hand massage has an effect on the pain levels of palliative cancer patients.
- **H**_**2**_**:** Hand massage has an effect on the sleep quality of palliative cancer patients.
- **H**_**3**_**:** Hand massage has an effect on the comfort of palliative cancer patients.

## Methods

### Study design and setting

**Fig. 1:**
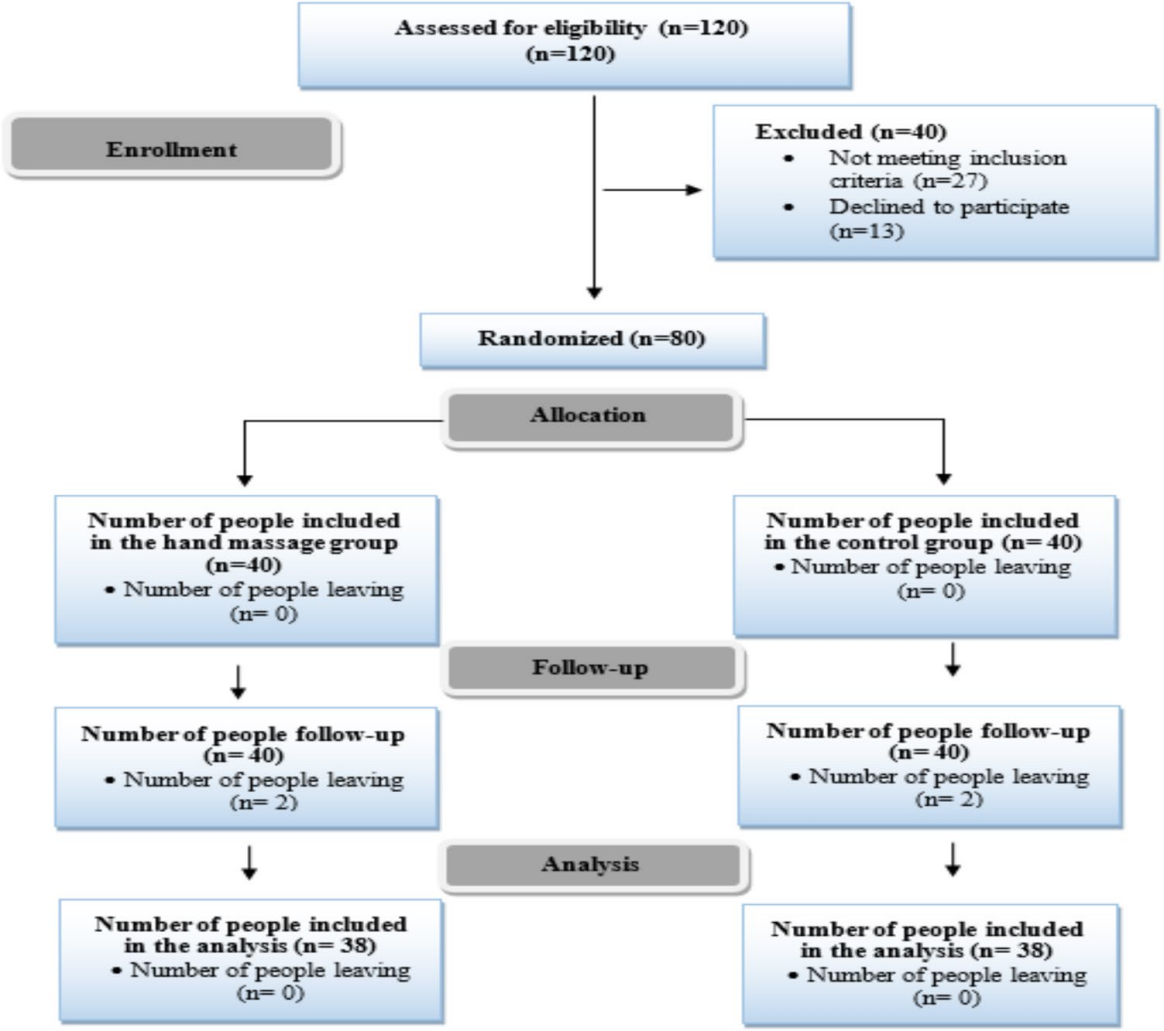
Flow diagram of the study participants (CONSORT 2020)

A two-group parallel randomized controlled trial was conducted with cancer patients being treated in the palliative care center of a public hospital in Istanbul between June 2024 and June 2025. The study was reported in accordance with the Consolidated Standards of Reporting Trials (CONSORT) guidelines (Fig. 1).

### Participants

The sample size for the study was determined with the G*Power 3.1.9.2 program based on Cohen’s standard effect sizes. To enable the detection of small differences, the effect size was accepted as 0.68, type I error as 5%, and power as 80% [26].

Inclusion criteria for the oncology patients in the study were: (1) treated as inpatients in the palliative care center for at least 2 weeks (2) completed oncological treatment, (3) at least 18 years of age, (4) conscious and cooperative with unimpaired verbal communication, (5) baseline visual analog scale (VAS) pain score above 3, (6) baseline Pittsburgh Sleep Quality Index (PSQI) score above 5, (7) no intravenous catheter in either hand, and (8) had a smartphone and agreed to wear a smart wristband during sleep.

Exclusion criteria were: (1) use of any analgesic drugs outside of the clinical protocol, (2) changes in the medical treatment protocol to address pain and sleep symptoms, (3) impaired tissue integrity in the hands, (4) diagnosed neurological problem, (5) loss to follow-up due to isolation, transfer, discharge, or death, and (6) not completing the data collection process for any reason.

The flow diagram of participant recruitment and group allocation is presented in Fig. 1. Initially, 120 patients were assessed for eligibility. Of these, 27 patients did not meet the inclusion criteria and 13 declined to participate; therefore, they were excluded prior to randomization. A total of 80 eligible patients were randomly allocated to the hand massage group (*n* = 40) or the control group (*n* = 40). Following randomization, two patients from each group were unable to complete the data collection process during the follow-up period and were excluded from the analysis. Consequently, statistical analyses were conducted on a total of 76 patients. The majority of the study sample consisted of oncology patients receiving palliative care during the advanced stages of the disease, reflecting an end-of-life care setting.

Statistical analyses were performed using a per-protocol approach, including only participants who completed all outcome assessments after randomization. An intention-to-treat analysis was not feasible because outcome data were entirely missing for participants lost to follow-up, precluding reliable data imputation.

### Randomization

Prior to group assignment, each participant was informed about the study and their written consent to participate was obtained. The study was conducted as a parallel trial with two groups, the experimental and control groups. Allocation was done by simple randomization using a random number table generated online (https://www.random.org). The numbers in this table were written on small, uniform pieces of paper, which were placed in an opaque box. Each patient was asked to draw a paper from the box, and the number written on the paper determined the participant's group assignment according to its counterpart in the randomization table. The randomization process was carried out by an independent nurse who was not involved in data collection or intervention delivery, thereby minimizing researcher involvement and ensuring allocation concealment. Due to the nature of the intervention and the need to perform the hand massage correctly according to protocol, the massage provider had to be aware of group assignments. Therefore, blinding could not be implemented.

### Data instruments

Data collection tools used in the study included a personal information form, patient follow-up form, the VAS for pain, the General Comfort Questionnaire (GCQ), the PSQI, and a smart wristband. The VAS, GCQ, and PSQI were administered at 4 time points: before the intervention and at 1 week, 2 weeks, and 4 weeks after the start of the intervention. To minimize respondent burden, the instruments were administered with researcher assistance and at appropriate times based on the participants’ clinical conditions.

#### Personal information form

This form was prepared by the researcher for the study. It consisted of 14 questions to determine the participants’ sociodemographic and disease characteristics (7 items), pain (3 items), and sleep characteristics (4 items).

#### Patient follow-up form

This form was prepared by the researcher and used to systematically record patient data including VAS pain, GCQ, and PSQI scores evaluated at baseline and at 1, 2, and 4 weeks, as well as sleep data obtained from the smart wristband.

#### Visual analog scale for pain

This 10-cm scale is commonly used to measure pain intensity.

#### General comfort questionnaire

This scale was developed to assess patients’ comfort level. The Turkish validity and reliability studies were conducted by Kuğuoğlu and Karabacak (2008). Scores range between 1 and 4, with a higher score indicating greater comfort. The scale’s Cronbach’s alpha coefficient of internal consistency reliability was reported as 0.88 [27].

#### Pittsburgh sleep quality index

The PSQI is a self-report questionnaire used to assess subjective sleep quality. The Turkish validity and reliability study was conducted by Ağargün et al. (1996). The scale is scored between 0 and 21, with scores ≥ 5 indicating poor subjective sleep quality. The Cronbach’s alpha coefficient was reported to be 0.80 [28].

#### Smart wristband (Objective Sleep Parameters)

A Xiaomi Smart Band 9 Pro smart wristband was used to monitor changes in objective sleep parameters, including sleep duration (hours) and device-generated sleep score. Sleep duration was categorized as follows: ≥ 7 h as good, 6–7 h as acceptable, 5–6 h as insufficient, and ≤ 5 h as poor sleep duration. The sleep score is expressed as a percentage, with a score of ≥ 85% indicating good, 70–84% moderate, and < 70% low sleep score. The device was used not for diagnostic purposes and was not considered a standalone measure of sleep quality, rather, it was used to monitor pre- and post-intervention changes in objective sleep patterns. Sleep scores were calculated automatically by the device using a proprietary algorithm incorporating parameters such as total sleep time, nighttime awakenings, and sleep stage estimation. As the algorithm is proprietary, detailed calculation procedures are not publicly available. Therefore, objective sleep data were interpreted in conjunction with subjective PSQI scores. Device calibration was performed monthly by an authorized service provider.

### Data collection

A pilot study was conducted with 10 patients to evaluate the comprehensibility of the scales and forms before use in the study sample. These data were not included in the analysis.

The study purpose and procedures were explained to patients who met the inclusion criteria, and written consent was obtained from those who agreed to participate. To eliminate interference between groups, both the experimental and control group patients stayed in single rooms throughout the intervention period.

Patients in the experimental group received two sessions of hand massage per day, twice weekly for four weeks, while patients in the control group received only routine follow-up and nursing care.

All patients in both groups were reassessed using the VAS, GCQ, and PSQI in face-to-face interviews held by the researcher 1 week, 2 weeks, and 4 weeks after the start of the intervention. Sleep duration and sleep score were measured using a smart wristband.

### Intervention group

Hand massage was performed on the patients in the experimental group by a member of the study team with relevant certification. This ensured standardized application of the intervention in accordance with the predefined protocol. The implementation of both the intervention and the assessment process by the same researcher was intended to ensure that the intervention protocol and the data collection procedures were applied in a standardized manner across all participants and to minimize variability related to different implementers. However, to reduce the potential risk of performance and detection bias associated with the researcher serving as both intervention provider and assessor, several measures were taken. All measurement instruments used in the study were structured and based on validated self-report scales. During the assessment process, the researcher read the scale items verbatim and in a neutral tone, refrained from providing explanatory or leading statements, and recorded the participants’ responses without interpretation, prompting, or feedback. The questions were administered solely for data collection purposes and not to confirm the effectiveness of the intervention. Participants were explicitly informed that their responses would not influence their care or the continuation of the intervention, and no additional comments or feedback regarding the intervention were provided. This approach was intended to preserve the objectivity of the evaluation process and reduce the likelihood of response bias related to researcher expectancy or social desirability.

Massages were based on the 10-min massage protocol used by Yücel and Eşer (2015) [29]. Although a standard protocol for hand massage interventions does not exist in the literature, previous studies have reported beneficial effects using varying frequencies and durations, indicating that flexible application schedules are common in clinical settings [20]. In our study, hand massage was administered twice a day on two days per week (Monday and Friday) for 4 weeks, resulting in a total of 16 sessions. This frequency and duration were considered sufficient to evaluate both immediate (acute) effects after each session and cumulative effects over time, while remaining feasible within routine clinical care. The intervention was scheduled for Mondays and Fridays at 2:00 pm and 8:00 pm. Mondays and Fridays were specifically chosen to integrate the sessions into the clinic’s workflow without disrupting routine activities and to allow for standardized assessment of outcomes across the week. Similarly, the times of 2:00 pm and 8:00 pm were selected to ensure that participants’ natural rest cycles were preserved and their essential care was not disrupted.

Before the intervention, the practitioner prepared for the massage, then informed the patient about the procedure and washed and dried the patient’s hands. The patient assumed a comfortable position with the hands supported by pillows or towels. Only an allowed patient companion was asked to remain in the patient room during the hand massage, which was performed using baby oil as a lubricant.

### Hand massage intervention

Hand massage was performed following the same steps on both hands. The hand and forearm muscles were first warmed with light strokes. The fingers were then individually stroked and gently compressed. Friction was applied to the back of the hand using medium pressure, and circular massage was applied to the area between the thumb and index finger. The wrist area was stimulated with small circular movements, and friction was applied to the palm using the thumbs. Then the finger and wrist joints were slowly loosened with rotational movements. The massage was concluded with light strokes to the back of the hand, and the same process was repeated on the other hand [30].

### Control group

Patients in the control group received routine follow-up and nursing care during the 4-week study period.

### Statistical analyses

IBM SPSS Statistics for Windows version 26.0 (IBM Corp., Armonk, NY, USA) package program was used to analyze the data obtained in the study. Number, percentage, mean, and standard deviation were used as descriptive statistics, and statistical comparisons were performed using independent samples t-test, chi-square test, Mann–Whitney U test, Fisher's exact test, one-way analysis of variance (ANOVA), and Bonferroni tests. All statistical tests were performed at a 95% confidence level and *p* < 0.05 was accepted for statistical significance.

### Ethical considerations

The study was conducted in accordance with the principles of the Declaration of Helsinki, with the approval of the ethics committee (date: 28.03.2024, decision no: 48) and the permission of the institution where the study was conducted. After being verbally informed about study purpose and procedures, all participants provided verbal and written informed consent. The study was registered at ClinicalTrials.gov (registration number: NCT06360614).

## Results

The mean age of the 76 participants was 52.59 ± 10.3 years. Most (85.5%) of the patients were married, 36% were elementary school graduates, and 23.7% had lung cancer (*p* > 0.05) (Table 1).

**Table 1:** Distribution of the sociodemographic and clinical characteristics of the cancer patients in the study groups (*N* = 76)

Characteristic		Experimental (*n* = 38)		Control (*n* = 38)		Total (*n* = 76)		Statistics
		n	%	n	%	n	%	
**Gender**	Female	18	47.4	17	44.7	35	46.1	χ^2^ = 0.05
	Male	20	52.6	21	55.3	41	53.9	p = 0.81
**Marital status**	Married	32	84.2	33	86.8	65	85.5	ET = 0.10
	Single	6	15.8	5	13.2	11	14.5	p = 0.74
**Education level**	Literate	11	29.7	6	15.8	17	22.7	ET = 2.93
	Elementary school	11	29.7	16	42.1	27	36.0	p = 0.56
	Middle school	10	27.0	9	23.7	19	25.3	
	High school	3	8.1	5	13.2	8	10.7	
	University	2	5.4	2	5.3	4	5.3	
**Diagnosis**	Lung cancer	8	21.1	10	26.3	18	23.7	χ^2^ = 0.88
	Colorectal cancer	7	18.4	9	23.7	16	21.1	p = 0.92
	Gastric cancer	9	23.7	7	18.4	16	21.1	
	Prostate cancer	8	21.1	7	18.4	15	19.7	
	Other	6	15.8	5	13.2	11	14.5	
		**Mean ± SD**		**Mean ± SD**		**Mean ± SD**		
**Age (years)**		51.16 ± 10.7		54.03 ± 9.9		52.59 ± 10.3		t = −1.21 p = 0.22
**Disease duration (years)**		3.5 ± 1.7		3.34 ± 1.51		3.42 ± 1.6		U = 698.0 p = 0.79
**Length stay in palliative care (months)**		12.14 ± 1.12		8.52 ± 1.86		10.33 ± 1.49		t = −1.10 p = 0.2

*t* Independent samples t-test, χ.^2^: Chi-square statistic, *ET* Fisher’s exact test statistic, *U* Mann-Whitney U test. *Significant (*p* < 0.05)

Comparison of VAS pain scores between the groups showed that the experimental group had significantly lower scores at 2 and 4 weeks compared to the control group (p < 0.05). Repeated measures analysis revealed no significant change in pain levels in the control group (p > 0.05), while the four measurements differed significantly in the experimental group (p < 0.001). In the experimental group, post-hoc analyses demonstrated significant reductions in pain at each follow-up compared to the previous measurement. At week 4, the mean VAS score was 2.76 ± 1.30 in the experimental group and 6.00 ± 1.30 in the control group, representing a mean difference of 3.24 points in favor of the experimental group (Table 2).

Comparisons of PSQI scores between the groups demonstrated better subjective sleep quality in the experimental group at 1, 2, and 4 weeks when compared with the control group (*p* < 0.05). There was no significant difference in repeated measures for the control group (*p* > 0.05). However, the experimental group showed a significant difference (*p* < 0.001), with subjective sleep quality improving at each follow-up compared to the previous measurement (*p* < 0.001). At week 4, the mean PSQI score was 12.97 ± 1.40 in the experimental group and 16.97 ± 1.88 in the control group, indicating a mean difference of 4.00 points (Table 2).

Comparison of the participants’ comfort scores between the groups showed that participants in the experimental group reported higher comfort levels than those in the control group at weeks 2 and 4 (*p* < 0.05). Consistent with this, no significant difference was found in repeated measures in the control group (*p* > 0.05). However, comfort scores in the experimental group differed significantly (*p* < 0.001), with greater comfort improving at each follow-up compared to the previous measurement (*p* < 0.001). At week 4, the mean comfort score was 2.67 ± 0.14 in the experimental group compared to 2.28 ± 0.12 in the control group, reflecting a mean difference of 0.39 points (Table 2).

**Table 2:** Comparison of pain, sleep quality, and comfort scores (*N* = 76)

Variable		Experimental Group (*n* = 38)	Control Group (*n* = 38)	Test statistics	Intergroup significance
**VAS**	**Pre**	7.34 ± 1.85	7.29 ± 1.96	t = 0.12	*p* = 0.90
	**Post W1**	6.00 ± 1.36	6.29 ± 1.33	t = −0.93	*p* = 0.35
	**Post W2**	4.42 ± 1.2	6.03 ± 1.13	t = −6.01 η^2^ = 0.32	*p* < 0.001* EG < CG*
	**Post W4**	2.76 ± 1.3	6.00 ± 1.3	t = −9.79 η^2^ = 0.56	*p* < 0.001* EG < CG*
	**Intragroup significance**	F = 127.47, *p* < 0.001* η^2^ = 0.84 Pre > W1,W2,W4* W1 > W2,W4* W2 > W4*	F = 13.57, p = 0.07		
**PSQI**	**Pre**	19.47 ± 1.37	19.53 ± 1.86	t = −0.14	*p* = 0.88
	**Post W1**	17.89 ± 1.45	18.79 ± 1.97	t = −2.25 η^2^ = 0.06	*p* < 0.001* EG < CG*
	**Post W2**	13.71 ± 1.29	17.87 ± 1.96	t = −10.91 η^2^ = 0.61	*p* < 0.001* EG < CG*
	**Post W4**	12.97 ± 1.4	16.97 ± 1.88	t = −10.50 η^2^ = 0.59	*p* < 0.001* EG < CG*
	**Intragroup significance**	F = 317.59, *p* < 0.001* η^2^ = 0.93 Pre > W1,W2,W4* W1 > W2,W4* W2 > W4*	F = 12.34, *p* = 0.21		
**GCQ**	**Pre**	2.22 ± 0.11	2.23 ± 0.14	t = −0.28	*p* = 0.77
	**Post W1**	2.29 ± 0.12	2.24 ± 0,13	t = 1.85	*p* = 0.06
	**Post W2**	2.65 ± 0.13	2.18 ± 0.12	t = 13.31 η^2^ = 0.7	*p* < 0.001* EG > CG*
	**Post W4**	2.67 ± 0.14	2.28 ± 0.12	t = 13.49 η^2^ = 0.71	p < 0.001* EG > CG*
	**Intragroup significance**	F = 217.59, p < 0.001* η^2^ = 0.90 Pre < W1,W2,W4* W1 < W2,W4*	F = 0.57, p = 0.63		

*VAS* Visual analog scale for pain, *PSQI* Pittsburgh Sleep Quality Index, *GCQ* General Comfort Questionnaire, *EG* Experimental group, *CG* Control group, *Pre* Pre-intervention, *Post* Post-intervention, *W* Week, *t* Independent samples t test, *F* Repeated measures ANOVA (Wilks' lambda), *Post-hoc* Bonferroni test, η^2^ Eta squared, *Significant (*p* < 0.05).

According to the data obtained from the smart wristbands, both objective sleep duration and device-generated sleep score were significantly higher in the experimental group than in the control group at 1, 2, and 4 weeks (p < 0.05). For both variables, repeated measures did not differ significantly in the control group (p > 0.05) but demonstrated significant improvement in the experimental group (p < 0.001). Objective sleep duration and device-generated sleep score increased significantly in the experimental group at week 2 and week 4 compared to baseline and week 1 (p < 0.001) (Table 3).

**Table 3:** Comparison of objective sleep parameters obtained with smart wristbands (N = 76)

Variable		Experimental Group (*n* = 38)	Control Group (*n* = 38)	Test statistics	Intergroup significance
**Sleep duration (hours) **	**Pre**	5.23 ± 0.4	5.7 ± 0.72	t = −1.88	*p* = 0.07
	**Post W1**	5.99 ± 0.31	5.37 ± 0.79	t = −4.52 η^2^ = 0.21	*p* < 0.001* EG > CG*
	**Post W2**	7.3 ± 0.41	6.05 ± 0.88	t = 7.97 η^2^ = 0.46	*p* < 0.001* EG > CG*
	**Post W4**	7.45 ± 0.56	6.2 ± 0.83	t = 7.69 η^2^ = 0.44	*p* < 0.001* EG > CG*
	**Intragroup significance**	F = 256.65, *p* < 0.001* η^2^ = 0.91 Pre > W2,W4* W1 > W2,W4*	F = 8.48, *p* = 0.09		
**Device-generated sleep score** **(%)**	**Pre**	65.76 ± 4.78	63.47 ± 6.09	t = −2.95	*p* = 0.13
	**Post W1**	67.26 ± 3.72	66.24 ± 5.83	t = −4.43 η^2^ = 0.21	*p* < 0.001* EG > CG*
	**Post W2**	81.87 ± 2.82	67.89 ± 7.52	t = 7.65 η^2^ = 0.44	*p* < 0.001* EG > CG*
	**Post W4**	82.95 ± 3.99	68.95 ± 6.54	t = 8.04 η^2^ = 0.46	*p* < 0.001* EG > CG*
	**Intragroup significance**	F = 175.33, *p* < 0.001* η^2^ = 0.88 Pre > W2,W4* W1 > W2,W4*	F = 5.31, *p* = 0.06		

*EG* Experimental group, *CG* Control group, *Pre* Pre-intervention, *Post* Post-intervention, *W* Week, *t* Independent samples t test, *F* Repeated measures ANOVA (Wilks' lambda), *Post-hoc* Bonferroni test, η^2^: Eta squared, *Significant (*p* < 0.05).

## Discussion

Our literature search yielded no previous study evaluating changes in pain, sleep quality, and comfort levels after a hand massage intervention in palliative oncology patients. Therefore, the results of the current study are discussed in comparison with the findings of studies examining the effect of hand massage on pain in other patient groups.

The results demonstrated that hand massage reduced pain in palliative oncology patients, supporting our first hypothesis. Similarly, hand massage has been reported to significantly reduce pain intensity in palliative care patients [17, 31], chemotherapy patients [25], surgical patients [32–34], people with rheumatoid arthritis [35], older adults with chronic pain [36], and liver transplant patients [37]. Hand massage was also reported to have an analgesic effect in patients undergoing transradial percutaneous coronary intervention [38]. In addition, the LI4 (He Gu) point, which anatomically corresponds to the area stimulated during hand massage, is recognized as one of the principal acupuncture points associated with pain relief [39]. In the literature, a meta-analysis including twenty-one studies demonstrated that acupuncture significantly reduced pain intensity in chemotherapy-induced peripheral neuropathy, and reported LI4 (He Gu) as one of the core acupuncture points associated with this analgesic effect [40]. In a comprehensive systematic review evaluating acupuncture and derived therapies applied to cancer patients receiving palliative care, a significant reduction in pain intensity was reported, and LI4 (Hegu) was emphasized as the most frequently used acupuncture point in these studies [41]. Our study provides additional evidence suggesting that hand massage may be a useful supportive method for pain management in palliative oncology patients.

The hand massage intervention was also associated with increased sleep quality among the palliative oncology patients in our study, supporting our second hypothesis. In another study of palliative oncology patients, Mercadante et al. (2015) determined that 60.8% of the patients had poor sleep quality [10]. Lee and Kim (2011) reported that hand massage increased sleep satisfaction and sleep duration in oncology patients [42]. Cho and Kim (2015) also found that hand massage increased sleep duration in patients presenting for orthopedic surgery [43]. Kudo and Sasaki (2020) found that hand massage increased sleep efficiency and reduced sleep onset latency in older women with sleep disorders [24]. Hand massage was also associated with improved sleep quality among hemodialysis patients in a study by Arslan and Akça (2020) [44] and in older women in a study by Seo and Chang (2009) [45]. Similar to the literature, the results of the present study indicate that hand massage is an effective nonpharmacological way to improve sleep quality. Considering the bidirectional relationship between pain and sleep, the significant reduction in pain observed in the intervention group may have contributed to the increase in total sleep duration and improvement in device-generated sleep score. Although causality cannot be established, these findings suggest that pain relief may represent one possible mechanism underlying the observed improvements in both subjective and objective sleep parameters.

We also observed that hand massage applied to palliative care oncology patients increased comfort levels, supporting our third hypothesis. Similar results have been obtained in different studies examining the effect of hand massage on comfort. Hand massage was reported to enhance comfort in patients scheduled for cataract surgery [22], older adults [46], and elderly nursing home residents [47–49]. Similar to the literature, the findings of our study suggest that hand massage may serve as a safe and supportive nonpharmacological approach to enhance comfort in palliative oncology patients.

### Study strengths

Methodological strengths of the study are the randomized controlled design, homogenous patient groups, and performance of hand massage by a certified practitioner according to a standardized protocol. In addition, sample size calculation and statistical analyses were conducted by an independent statistician, enhancing methodological rigor.

### Study limitations

The main limitations of the study are that it was conducted within a specific time period and was limited to the oncology patients treated at a single palliative care center. Moreover, the fact that the study was conducted in a public hospital also limits the generalizability of the results. Furthermore, the absence of patients with breast and gynecological oncology diagnoses in the study sample limits the applicability and replicability of the results to these diagnostic groups. Additionally, the use of a per-protocol approach represents another limitation, as it may have reduced the benefits of randomization and introduced attrition-related bias. Finally, the same researcher conducted both the intervention and outcome assessments, which may represent a potential risk of measurement bias despite the use of structured and validated instruments and efforts to standardize the assessment process.

## Conclusion

Hand massage was associated with reduced pain intensity, improved subjective sleep quality, enhanced objective sleep parameters (sleep duration and device-generated sleep score), and increased comfort. These findings suggest that hand massage may be considered as a supportive, low-cost nonpharmacological intervention in palliative oncology settings. Future studies with larger and more diverse samples are needed to confirm these findings.

## Data Availability

The data that support the findings of this study are available from the corresponding author, [NYA], upon reasonable request.

## Declarations

### Permission to reproduce material from other sources

No material for which copy permission is required was not used.
